# Supplementary material for: The Evolution and Characterization of the RNA Interference Pathways in Lophotrochozoa
Source: Genome Biol Evol. 2024 May 7;16(5):evae098. doi: 10.1093/gbe/evae098 (PMC11114477; doi:10.1093/gbe/evae098)
Supplement: evae098_Supplementary_Data [file evae098_supplementary_data.zip › Supplementary_materials.zip!/selected_miRNAs/NoGe_selected_miRNAs/known_miRNA_families_pdf_files/NMRB01000152.1_Notospermus_geniculatus_strain_Ushimado_scaffold152,_whole_genome_shotgun_sequence_5848.pdf]

Provisional ID	:	NMRB01000152.1_Notospermus_geniculatus_strain_Ushimado_scaffold152,_whole_genome_shotgun_sequence_5848
Score total	:	3.6
Score for star read(s)	:	-1.3
Score for read counts	:	0
Score for mfe	:	1.9
Score for randfold	:	0
Score for cons. seed	:	3
Total read count	:	35072
Mature read count	:	19386
Loop read count	:	0
Star read count	:	15686

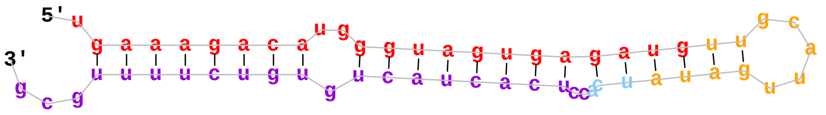

Genome coordinates: NMRB01000152.1 418129-418188

pre-miRNA sequence:
UGAAAGACAUGGGUAGUGAGAUGUGAAAGACAUGGGUAGUGAGAUGUUGCAUUGAUAUCACCUCACUACUGUGUCUUUUGCG

Mature sequence: UGAAAGACAUGGGUAGUGAGAUG

Aligned mature sequence of the *N. geniculatus* pre-miRNA with the top 5 hits from the BLAST search against the pre-miRNA mirGeneDB set:

```
                        ***********************
Nge_Mir-71-P5           ugaaagacaugggguagugagaug
Bge-Mir-71_5p           ugaaagacaugggguagugagaug
Lan-Mir-71-P4_5p        ugaaagacaugggguagugagaug
Lan-Mir-71-P5_5p        ugaaagacaugggguagugagaug
```
